# Surgical treatment of esophago-tracheobronchial fistulas after esophagectomy

**DOI:** 10.1093/dote/doad054

**Published:** 2023-08-17

**Authors:** E M de Groot, B F Kingma, L Goense, N P van der Kaaij, R C A Meijer, F Z Ramjankhan, P A A Schellekens, S A Braithwaite, M Marsman, J J van der Heijden, J P Ruurda, R van Hillegersberg

**Affiliations:** Department of Surgery, University Medical Center Utrecht, Utrecht, The Netherlands; Department of Surgery, University Medical Center Utrecht, Utrecht, The Netherlands; Department of Surgery, University Medical Center Utrecht, Utrecht, The Netherlands; Department of Cardiothoracic Surgery, University Medical Center Utrecht, Utrecht, The Netherlands; Department of Cardiothoracic Surgery, University Medical Center Utrecht, Utrecht, The Netherlands; Department of Cardiothoracic Surgery, University Medical Center Utrecht, Utrecht, The Netherlands; Department of Plastic Surgery, University Medical Center Utrecht, Utrecht, The Netherlands; Department of Anesthesiology, University Medical Center Utrecht, Utrecht, The Netherlands; Department of Anesthesiology, University Medical Center Utrecht, Utrecht, The Netherlands; Department of Intensive Care, University Medical Center Utrecht, Utrecht, The Netherlands; Department of Surgery, University Medical Center Utrecht, Utrecht, The Netherlands; Department of Surgery, University Medical Center Utrecht, Utrecht, The Netherlands

**Keywords:** esophagectomy, tracheoesophageal fistula, treatment

## Abstract

The aim of this study was to evaluate the surgical treatment of esophago-tracheobronchial fistulas (ETBFs) that occurred after esophagectomy with gastric conduit reconstruction in a tertiary referral center for esophageal surgery. All patients who underwent surgical repair for an ETBF after esophagectomy with gastric conduit reconstruction were included in a tertiary referral center. The primary outcome was successful recovery after surgical treatment for ETBF, defined as a patent airway at 90 days after the surgical fistula repair. Secondary outcomes were details on the clinical presentation, diagnostics, and postoperative course after fistula repair. Between 2007 and 2022, 14 patients who underwent surgical repair for an ETBF were included. Out of 14 patients, 9 had undergone esophagectomy with cervical anastomosis and 5 esophagectomy with intrathoracic anastomosis after which 13 patients had developed anastomotic leakage. Surgical treatment consisted of thoracotomy to cover the defect with a pericardial patch and intercostal flap in 11 patients, a patch without interposition of healthy tissue in 1 patient, and fistula repair via cervical incision with only a pectoral muscle flap in 2 patients. After surgical treatment, 12 patients recovered (86%). Mortality occurred in two patients (14%) due to multiple organ failure. This study evaluated the techniques and outcomes of surgical repair of ETBFs following esophagectomy with gastric conduit reconstruction in 14 patients. Treatment was successful in 12 patients (86%) and generally consisted of thoracotomy and coverage of the defect with a bovine pericardial patch followed by interposition with an intercostal muscle.

## INTRODUCTION

Esophago-tracheobronchial fistula (ETBF) is a severe complication after esophagectomy with a reported incidence up to 3%.^1^ The predominant pathophysiology of ETBF is an ongoing infection between the esophagus and airway, mostly caused by anastomotic leakage of the esophagogastric anastomosis or longitudinal staple line of the gastric conduit.^2^^,^^3^ Patients with ETBF generally require prolonged intensive care unit (ICU) stay, and mortality rates up to 60% have been reported.^4^

To date, evidence on the most effective surgical strategy is limited and mainly consists of case-reports and a few case-series.^5^ Identification of the most effective treatment strategy is essential to optimize the chance of recovery from ETBF following esophagectomy. Reported treatment strategies can generally be classified in two broad categories: surgical repair and non-surgical repair.^5^ Several techniques are reported for surgical ETBF repair including a pericardial patch (both bovine and autologous) followed by the interposition of a muscle, muscle flap repair alone, and primary airway repair. Non-surgical treatment mainly involves stent placement. A recent study demonstrated that surgical ETBF repair was associated with superior survival rates compared to non-surgical treatment strategies.^5^ It must be noted that selection bias plays an important role in these studies as patients should be fit enough to undergo surgical fistula repair. Even though surgical ETBF repair seems to be associated with better survival, it remains a highly complex procedure that is still associated with high mortality rates.^6^

The aim of this study was to evaluate the surgical treatment of ETBF that occurred after esophagectomy with gastric conduit reconstruction in a tertiary referral center for esophageal surgery.

## METHODS

### Study population

Patients who underwent surgical repair for ETBF between 2007 and 2022 in the University Medical Center Utrecht (UMC Utrecht) were identified from the patients’ medical record via an automated query in the electronic health record. All patients who underwent surgical repair for ETBF which developed after esophagectomy with gastric conduit reconstruction were included. There were no further exclusion criteria. The ethical board approved this study and informed consent was waived (document number 22–867).

### ETBF

Details on ETBF were retrospectively extracted from the patient medical records. ETBF was defined by a fistula between the gastric conduit and the bronchus or trachea as confirmed intraoperatively. Cough, pneumonia, and air leak were considered symptoms suspected for ETBF. Computed tomography (CT) scans and bronchoscopy reports were evaluated to determine whether the fistula was visible or not. Surgical reports were reviewed to determine the exact location of the fistula.

### Surgical fistula repair

**Fig. 1:**
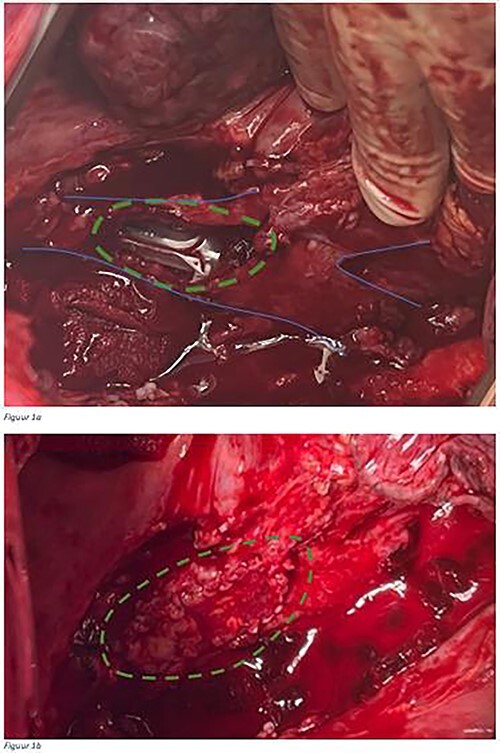
An esophago-tracheobronchial fistula in a patient with a large defect ( dotted line) in the trachea ( line) after the gastric conduit was removed (a). Esophago-tracheobronchial fistula repair with a bovine patch that was attached by a running suture (b).

**Fig. 2:**
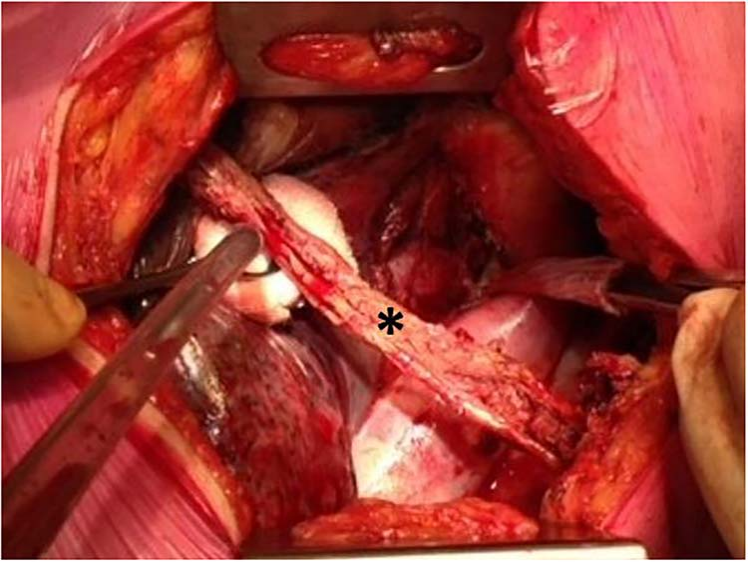
Harvest of an intercostal muscle flap to cover the patch on the esophago-tracheobronchial fistula during thoracotomy. The intercostal flap is marked with a ^*^.

Surgical repair of ETBF in the UMC Utrecht is based on three pillars. First, in the majority of cases, the fistula is caused by ongoing, inadequately drained anastomotic leakage, which results in abscess formation as a source of the fistula. This leak needs to be controlled by endoscopic or surgical drainage and if necessary, the gastric conduit has to be disconnected to avoid recurrence of the fistula. Disconnection of the gastric conduit consists of disconnecting the anastomosis, resecting the gastric conduit, and generally creating a cervical esophagostomy. Another reason to disconnect the gastric conduit is to create adequate surgical exposure of the ETBF. Second, the ETBF should be surgically repaired airtight by covering the airway defect with a bovine or an autologous pericardial patch (Fig. 1). Last, this patch should preferably be covered with healthy tissue, for example an intercostal muscle flap, which concludes the procedure (Fig. 2).

Peri- and postoperatively, ventilation pressures should be minimized in order to protect the patch. Preferably, patients should breath spontaneously. If the patient requires ventilation and the tube is reaching and covering the bovine patch, a tracheostomy may be considered to avoid damage to the patch. If the fistula is in the left main bronchus, extracorporeal membrane oxygenation (ECMO) may be necessary to provide oxygenation and prevent hypercapnia without lung ventilation, while repairing the defect through a right thoracotomy. In general, venovenous ECMO (VV-ECMO) is sufficient to reach adequate oxygenation.

### Outcomes

The primary outcome was successful recovery after surgical treatment for ETBF defined as a patent airway at 90 days after the surgical fistula repair. Secondary outcomes were: days between esophagectomy and diagnosis of ETBF, whether the fistula was visible on CT-scan and bronchoscopy, intraoperative ventilation technique, postoperative complications after the fistula repair including pneumonia and recurrent laryngeal nerve palsy, in-hospital mortality, 90-day mortality after ETBF repair, duration of hospital stay measured from fistula repair to discharge, and duration of ICU stay after ETBF repair.

### Statistical analysis

Only descriptive analysis was performed. Categorical variables were reported as a number with percentage. Continuous variables were reported as mean with standard deviation or a median with range, pending on data distribution. All statistical analyses were performed by using SPSS 25.0 (IBM).

## RESULTS

### Patient characteristics

Between 2007 and 2022, 14 patients underwent surgical repair for ETBF and were included in the study. Patient and treatment characteristics are demonstrated in Table 1. Before the ETBF developed, all patients underwent an esophagectomy with gastric conduit reconstruction as treatment for esophageal cancer. Most patients had undergone neoadjuvant therapy (*n* = 11) followed by esophagectomy with a cervical anastomosis (*n* = 9) or an intrathoracic anastomosis (*n* = 5). Anastomotic leakage was diagnosed in 13 of the 14 included patients. The only patient whom developed ETBF without being preceded by anastomotic leakage suffered from an iatrogenic esophageal perforation during endoscopic dilatation of an anastomotic stricture.

**Table 1:** Patient and treatment characteristics of 14 patients with an ETBF developed after esophagectomy with gastric conduit reconstruction

Variable	Patients (*n* = 14)
**Sex** Male Female	10 (71) 4 (29)
**Age**, median (range)	64 (49–75)
**Tumor histology** Adenocarcinoma Squamous cell carcinoma Missing	9 (64) 4 (29) 1 (7)
**pTNM stage** Complete regression T2N0M0 T2N1M0 T1N0M0 Missing	6 (43) 4 (29) 1 (7) 1 (7) 2 (14)
**Neoadjuvant treatment** Chemoradiotherapy None Missing	11 (79) 1 (7) 2 (14)
**Esophagectomy** Ivor-Lewis McKeown Transhiatal	5 (36) 7 (50) 2 (14)
**Anastomotic leakage after esophagectomy**	13 (86)

### Presentation and diagnosis

**Fig. 3:**
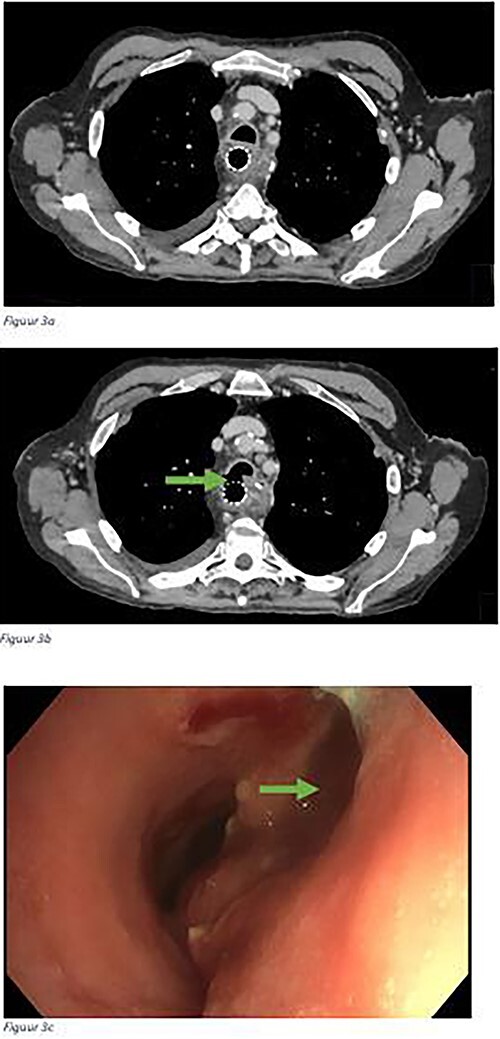
A CT-scan of a patient with an esophago-tracheobronchial fistula with a stent in the gastric conduit and patent airway in (a) and a visible defect in the trachea on (b) which was also observed during bronchoscopy (c). The arrow is pointing to the esophago-tracheobronchial fistula.

Details on ETBF are presented in Table 2. The ETBF was diagnosed after a median of 2 months (range 0–42 months) following esophagectomy. Patients presented with aspiration pneumonia(s) (*n* = 5), cough (*n* = 4), air leak trough the neck wound or gastric tube (*n* = 2), or as coincidental finding during endoscopy for dilatations (*n* = 2). A bronchoscopy was performed in 12 patients in which the fistula was visible in 10 patients. Of the 14 patients, 12 patients underwent a CT scan during the diagnostic workup. In 7 of these 12 patients, the fistula was visible on the CT scan. The ETBF was located in the trachea in six patients (43%), the right main bronchus in five patients (36%) and in the left main bronchus in two patients (14%). An example of an ETBF located at the trachea is demonstrated in Figure 3.

**Table 2:** Details on the ETBF of 14 patients after esophagectomy with gastric conduit reconstruction

Variable	Patients (*n* = 14)
**Diagnosis after esophagectomy, days, median (range)**	72 (17–1291)
**Symptoms** Cough Aspiration pneumonia Airleak (neck wound/gastric tube) Coincidental finding Unknown	4 (29) 5 (36) 2 (14) 2 (14) 1 (7)
**Location fistula** Trachea Right main bronchus Left main bronchus	6 (43) 6 (43) 2 (14)
**Fistula visible on bronchoscopy** Yes No Not performed	10 (72) 2 (14) 2 (14)
**Fistula visible on CT-scan** Yes No Not performed	7 (50) 5 (36) 2 (14)
**Timing fistula repair** During postoperative course after esophagectomy During follow-up	5 (36) 9 (64)

### Surgical fistula repair

**Fig. 4:**
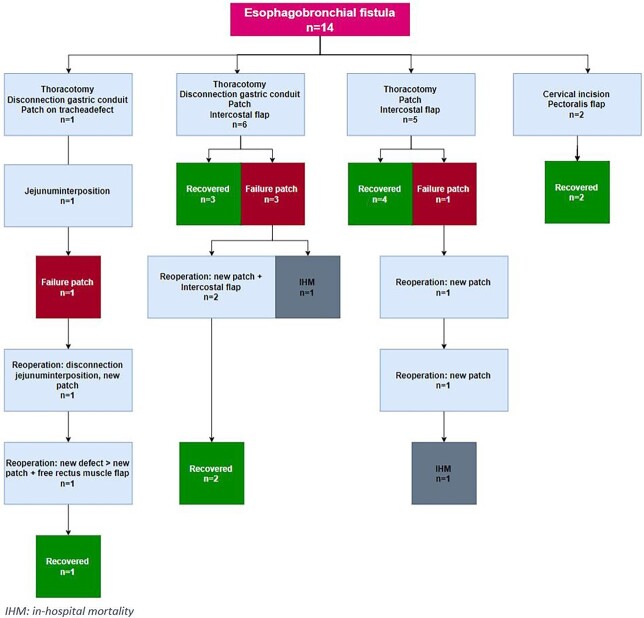
Evaluation of the management of 14 patients with an esophagobronchial fistula developed after esophagectomy with gastric conduit reconstruction.

Surgical repair of ETBF was performed after a median of 36 (range 0–788) days following diagnosis. Overall, surgical treatment of ETBF was successful in 12 patients (86%). Details of the surgical fistula repair and its postoperative course are demonstrated in Figure 4.

Eleven of 14 patients (79%) underwent surgical repair with a pericardial patch (bovine *n* = 8, autologous *n* = 3) covered with an intercostal muscle flap. Out of these 11 patients who received a pericardial patch and an intercostal muscle flap, nine patients ultimately recovered successfully. Two patients required a reoperation due to patch detachment. Both reoperations involved repair with a new patch (bovine *n* = 1, autologous *n* = 1) and coverage with an intercostal muscle flap, after which recovery was successful. Two of the 11 patients (14%) died. Both patients died of multiple organ failure following patch detachment, 9 and 7 days after fistula repair, respectively.

Two out of 14 patients (14%) who previously underwent McKeown esophagectomy underwent fistula repair via a cervical incision and coverage of the defect with a pectoralis muscle flap. In these patients, no patch was used. Both patients recovered successfully.

One out of the 14 patients underwent fistula repair via a thoracotomy followed by a disconnection of the gastric tube and bovine patch placement on the airway defect. After 3 days, restoration of the gastrointestinal tract was performed with a jejunum interposition. The patient developed anastomotic leakage of the jejunum interposition after which the bovine patched detached. A reoperation was required to disconnect the jejunum interposition and cover the ETBF with a new patch. During the postoperative course, a new airway defect developed right under the patch requiring a reoperation to cover the defect with a new patch, which was covered with a free rectus muscle flap after which the patient recovered successfully.

### Intraoperative ventilation

Intraoperative ventilation consisted of left-sided single lung ventilation in the majority of the patients (*n* = 8), who underwent a right-sided thoracotomy. Single lung ventilation was achieved with a double lumen tube in seven patients and a bronchus blocker in one patient. ECMO was used in three patients, of which one patient had an ETBF located at the left main bronchus, one patient at the trachea, and one patient had a history of a left lung lobectomy. In one patient, a left-sided thoracotomy was performed requiring right-sided single-lung ventilation with a double lumen tube as the defect was located at the left main bronchus. A thoracotomy and consequently single-lung ventilation could be avoided in two patients who underwent surgical fistula repair via cervical access.

### Postoperative outcomes

Postoperative outcomes after surgical repair of ETBF are presented in Table 3. Of 14 patients who underwent surgical fistula repair, five patients (36%) developed a pneumonia and four patients (29%) a laryngeal recurrent nerve palsy. Reoperations were required in seven patients (50% and included replacement of the detached patch (*n* = 5), thoracic drainage because of anastomotic leakage (*n* = 1) and a surgical tracheostomy (*n* = 1). Patch detachment itself was a reason to return to the OR. In total, a tracheostomy was performed in five patients (36%) of which four were placed during the primary fistula repair intervention. Median hospital stay was 21 days (range 7–105), and the median stay on the ICU was 7 days (range 0–57). In-hospital mortality and 90-day mortality were 14% (*n* = 2). In seven patients (50%), the gastric conduit was disconnected and resected during the fistula repair surgery. The reason for conduit resection is to create source control of the leakage in case the conduit tissue is in a bad condition, either inflamed or stenotic, which makes primary repair impossible. Restoration of the gastrointestinal tract with a jejunum or colon interposition was performed in four patients; simultaneously with the fistula repair surgery in one patient, 3 days after the fistula repair surgery in one patient and at a later stage in the other two patients. The other three patients did not undergo restoration of the gastrointestinal tract because of mortality (*n* = 1), development of recurrent esophageal cancer (*n* = 1), and one patient is currently waiting for restoration.

**Table 3:** Postoperative outcomes after surgical repair of an ETBF developed after esophagectomy with gastric conduit reconstruction in 14 patients

Variable	Patients (*n* = 14)
**Complications** Pneumonia Recurrent nerve palsy	5 (36) 4 (29)
**Reoperation(s)** Esophagobronchial fistula related Anastomotic leakage Tracheostomy None	5 (36) 1 (7) 5 (36) 7 (50)
**Hospital stay, days, median (range)**	21 (7–105)
**ICU stay, days, median (range)**	7 (0–57)
**In-hospital mortality**	2 (14)
**90-day mortality**	2 (14)

## COMMENT

This study evaluated the surgical repair of ETBF, which developed after esophagectomy with gastric conduit reconstruction in 14 patients. To our knowledge, this is one of the largest reports on this topic. To date, only case reports and few case series were published on the surgical management of ETBF following esophagectomy.^2^^,^^7^ Previous studies reported mortality rates up to 60% after surgical repair, mainly caused by respiratory failure, sepsis, and multiple organ failure.^6^ In the current study, the operative mortality and 90-day mortality rate were 14% which is considerably lower, supporting the management strategies used in this study.

Surgical management of ETBF consisted of three principles. First, in case the patient simultaneously suffers from anastomotic leakage, the leak should be adequately drained to create source control. If necessary, the gastric conduit should be disconnected in order to create adequate surgical exposure and achieve source control. Second, an air tight reconstruction that would also withstand mechanical ventilation with positive air pressure should be applied (i.e. a bovine patch). Third, interposition with healthy tissue, preferably an intercostal muscle, to allow healing of the defect concludes the procedure. The success of interposition of healthy tissue using a muscle flap has been demonstrated previously in relatively large case series on ETBF.^8^^,^^9^ However, in these studies, ETBF with all kind of etiologies were included and only few patients who developed an ETBF after esophagectomy were involved. ETBF after esophagectomy requires a different approach because these patients often have a difficult accessible thorax due to the effects of anastomotic leakage and previous radiation and surgery. In the literature, only few alternatives are reported for surgical ETBF repair, including primary closure of the airway defect with stitches alone. This technique is only possible for small defects and for patients without simultaneous anastomotic leakage. In addition, primary closure results in some narrowing of the airway, and it should be anticipated if a patient can tolerate this.^6^ Another reported alternative is tracheal resection with reconstruction. However, also these papers generally included patients with ETBF with a different etiology than post-esophagectomy.^9^^,^^10^ A recent study published on surgical repair of ETBF after esophagectomy which consisted of primary suturing followed by coverage with a muscle flap achieved a survival rate of 63%.^11^In our opinion, it is difficult to attain an air tight repair of the airway with only a muscle flap especially if the patient needs postoperative mechanical ventilation with positive pressure on the reconstruction in an inflamed mediastinum. In our institution, we use a bovine patch for every fistula regardless of the size, as this patch is very strong so tension can be put on the patch to prevent bulging in the airway and this can be attached air tight.

Although in this study a relatively low mortality rate was achieved compared to previous series, ETBF was associated with considerable morbidity in this study. This is demonstrated by an overall complication rate of 79% and patch detachment in five patients (42%). In four of these patients, the fistula repair surgery took place during the postoperative course after esophagectomy, which was complicated by anastomotic leakage. The other patient had a similar situation as the patient underwent a jejunum interposition 3 days after the fistula repair. The patient developed leakage of the jejunum interposition and subsequently the patch detached. It is hypothesized that these five patients were in a catabolic and septic state, which impeded the patch from healing, despite the disconnection of the gastric conduit in three patients. It is challenging to treat patients for ETBF while suffering from severe anastomotic leakage as it is generally not possible to postpone the fistula repair because these patients are in respiratory distress. Therefore, it is important to always manage the anastomotic leakage, which sometimes means disconnecting the gastric conduit. When the gastric conduit is disconnected, restoration of the gastrointestinal tract should be delayed until the ETBF is fully recovered and the patient is fit for subsequent surgery.

Although the current results show that surgical treatment of ETBF is challenging and frequently involves a second or even third operation due to inadequate primary patch ingrowth, it is important to realize that inadequate primary patch ingrowth does not necessarily indicate failure to rescue. This is demonstrated by the results of this study, which showed that three out of five patients in whom the primary patch detached were able to recover after further treatment.

Intra- and postoperative ventilation strategy is dependent on the location of the fistula and can be difficult during ETBF repair, because positive pressure ventilation impairs wound healing and therefore pressures should be kept as low as possible. In general, patients already are in a septic status, making low pressure ventilation or single lung ventilation challenging due to high oxygenation and ventilation need. If single lung ventilation is not possible during ETBF repair, VV-ECMO is a realistic alternative. However, a major disadvantage of VV-ECMO is the necessity of anticoagulation, potentially causing diffuse bleeding during the mediastinal dissection phase. If ECMO is required intraoperatively, it might be wise to use ECMO the first few days postoperatively as well, in order to reduce the ventilation pressure on the patch. In line with this theory, the patient should be extubated as soon as possible.

A strength of this study is that it is currently the largest case series on surgical treatment of ETBF after esophagectomy. In addition, the study population is homogenous as all patients underwent esophagectomy followed with gastric conduit reconstruction before the fistula developed. This study has also several limitations. First, this is a retrospective study in which most of the data were collected from the patients’ medical record. Second, patients with ETBF who did not underwent surgical repair were not included in this study, which might have been interesting to evaluate as well. Therefore, it is not possible to conclude if surgical repair is superior over non-surgical treatment for this patient cohort.

In conclusion, this single-center study evaluated the techniques and outcomes of surgical repair of ETBF following esophagectomy with gastric conduit reconstruction in 14 patients. Treatment generally consisted of thoracotomy and coverage of the defect with a bovine patch followed by interposition with an intercostal muscle. Although failure of primary patch ingrowth was relatively frequent (42%), this study showed that this does not necessarily indicate failure to rescue since most of these patients recovered after further treatment involving one or more re-operation(s). With an 86% final recovery rate, thoracotomy and airway repair with a bovine patch that is covered by an intercostal muscle may be advised as the first line of surgical treatment for ETBF after esophagectomy.
